# A novel methodology for in vivo endoscopic phenotyping of colorectal cancer based on real-time analysis of the mucosal lipidome: a prospective observational study of the iKnife

**DOI:** 10.1007/s00464-016-5121-5

**Published:** 2016-08-08

**Authors:** James Alexander, Louise Gildea, Julia Balog, Abigail Speller, James McKenzie, Laura Muirhead, Alasdair Scott, Christos Kontovounisios, Shanawaz Rasheed, Julian Teare, Jonathan Hoare, Kirill Veselkov, Robert Goldin, Paris Tekkis, Ara Darzi, Jeremy Nicholson, James Kinross, Zoltan Takats

**Affiliations:** 1 Department of Surgery and Cancer, Imperial College London, St. Mary’s Hospital, Praed Street, London, NW1 1SQ UK; 2 Waters Corporation, Wilmslow, UK; 3 Department of Histopathology, Imperial College London, London, UK; 4 Department of Gastroenterology, Imperial Healthcare NHS Trust, London, UK; 5 Department of Colorectal Surgery, Royal Marsden Hospital NHS Trust, London, UK

**Keywords:** Rapid evaporative ionization mass spectrometry, Colorectal cancer, Metabolic

## Abstract

**Background:**

This pilot study assessed the diagnostic accuracy of rapid evaporative ionization mass spectrometry (REIMS) in colorectal cancer (CRC) and colonic adenomas.

**Methods:**

Patients undergoing elective surgical resection for CRC were recruited at St. Mary’s Hospital London and The Royal Marsden Hospital, UK. Ex vivo analysis was performed using a standard electrosurgery handpiece with aspiration of the electrosurgical aerosol to a Xevo G2-S iKnife QTof mass spectrometer (Waters Corporation). Histological examination was performed for validation purposes. Multivariate analysis was performed using principal component analysis and linear discriminant analysis in Matlab 2015a (Mathworks, Natick, MA). A modified REIMS endoscopic snare was developed (Medwork) and used prospectively in five patients to assess its feasibility during hot snare polypectomy.

**Results:**

Twenty-eight patients were recruited (12 males, median age 71, range 35–89). REIMS was able to reliably distinguish between cancer and normal adjacent mucosa (NAM) (AUC 0.96) and between NAM and adenoma (AUC 0.99). It had an overall accuracy of 94.4 % for the detection of cancer versus adenoma and an adenoma sensitivity of 78.6 % and specificity of 97.3 % (AUC 0.99) versus cancer. Long-chain phosphatidylserines (e.g., PS 22:0) and bacterial phosphatidylglycerols were over-expressed on cancer samples, while NAM was defined by raised plasmalogens and triacylglycerols expression and adenomas demonstrated an over-expression of ceramides. REIMS was able to classify samples according to tumor differentiation, tumor budding, lymphovascular invasion, extramural vascular invasion and lymph node micrometastases (AUC’s 0.88, 0.87, 0.83, 0.81 and 0.81, respectively). During endoscopic deployment, colonoscopic REIMS was able to detect target lipid species such as ceramides during hot snare polypectomy.

**Conclusion:**

REIMS demonstrates high diagnostic accuracy for tumor type and for established histological features of poor prognostic outcome in CRC based on a multivariate analysis of the mucosal lipidome. REIMS could augment endoscopic and imaging technologies for precision phenotyping of colorectal cancer.

**Electronic supplementary material:**

The online version of this article (doi:10.1007/s00464-016-5121-5) contains supplementary material, which is available to authorized users.

Precision medicine aims to improve patient outcomes, by coupling established clinical–pathological indexes with state-of-the-art molecular profiling to create diagnostic, prognostic and therapeutic strategies precisely tailored to each patient’s requirements [1, 2]. There is an unmet need for precision polyp and cancer phenotyping for stratification of surgical and neoadjuvant therapies in the treatment of colorectal cancer. In the management of complex rectal polyps and early rectal cancer, the decision to proceed to a radical resection is largely based on a combination of imaging and clinical assessment. Current protocols for risk stratification of polyps are based on subjective descriptions of polyp morphology such as the Paris classification although these are not highly reproducible [3] and histopathological analysis is also subjective.

Rapid evaporative ionization mass spectrometry (REIMS) is a novel technique based on the mass spectrometric analysis of the aerosol produced during electrosurgical diathermy to provide tissue specific diagnostics based on lipid profiles [4–6]. This technology is able to provide rapid metabolic phenotyping of rectal and colonic cancers using the mass spectrometric lipid signatures for the identification of unknown tissue features intra-operatively with correct classification efficiencies exceeding 92 % in real-time and 97 % for ex vivo analyses [7]. Recently, we have demonstrated that this technology can be deployed endoluminally in the upper and lower GI tract; however, its true application in colonoscopy is not yet known [8]. As a follow-up to our earlier work, in this pilot study, we assessed the accuracy of endoscopic REIMS for the classification of both benign and neoplastic colorectal lesions and to provide chemical information associated with poor prognostic histological features commonly assessed in colorectal cancer. The aim was to assess the feasibility of this technology to provide information of clinical utility, and this was achieved using an ex vivo analysis of tissue samples, which permits the creation of a spectral database for prospective use. The secondary aim was to further demonstrate that in vivo applications of endoscopic REIMS could be scaled and reliably reproduced. A prototype was therefore developed for deployment of REIMS during colonoscopy.

## Methods

### Patient recruitment and sampling

Between January and March 2015, 28 patients undergoing elective surgical treatment for colorectal cancer were prospectively recruited at two London teaching hospitals (St Mary’s Hospital and The Royal Marsden Hospital). Full local ethical approval was obtained (14/EE/0024). Inclusion criteria were adult patients with a suspected primary colorectal cancer undergoing elective surgical resection by either open or laparoscopic surgery. Patients who had undergone neoadjuvant chemoradiotherapy were included. Patients unable to provide consent or those undergoing emergency surgery were excluded, as were patients with tumors associated with hereditary conditions such as Familial Adenomatous Polyposis, Hereditary Nonpolyposis Colorectal Cancer or inflammatory bowel disease. The primary endpoint of the study was the diagnosis of colorectal cancer, as differentiated from adenoma and normal adjacent mucosa (NAM). Secondary endpoints were the diagnosis of histological prognostic biomarkers: TNM stage, tumor differentiation, lymphovascular invasion, extramural vascular invasion and tumor budding and complete pathological response (cPR) to neoadjuvant therapy. Following resection, surgical specimens were immediately transported fresh to the histopathology department by a member of the research team. The specimen was opened by a trained histopathologist, and fresh biopsies were cut from the tumor and normal adjacent mucosa at 10 cm from the lesion.

### Ex vivo analysis

The fresh biopsies were analyzed ex vivo with an electrosurgery hand-piece using monopolar diathermy in cutting mode (ValleylabTM) with aspiration of the electrosurgical aerosol to a Xevo G2-S iKnife Q-Tof mass spectrometer (Waters Corporation). Following formalin fixation, the biopsies underwent histological examination by two trained histopathologists (AS & RG) for validation purposes. Full classification was performed according to the minimum dataset recommendations of the Royal College of Pathologists [9].

### Statistical analysis

Four high-quality mass spectra were obtained per sample. REIMS raw spectral data were converted to imzML format (MSConvert) and imported into Matlab R2015a (Mathworks, Natick, MA) for processing within a toolbox designed in-house for interrogation of clinical mass spectral datasets. The compiled spectra were normalized to their total ion count to reduce the impact of variation in overall signal intensity unrelated to spectral pattern and then log transformed to account for large variability differences between small and large peaks across mass spectra [10]. Discriminating models were then subjected to dimensionality reduction via principal components analysis to reduce the number of observed variables into a smaller number of orthogonal principal components, which account for the majority of the variance in the dataset. The first 30 principal components were kept for further analysis. Linear discriminant analysis was then used to maximize the separation between different classes, and the resulting LDA space was used for classification of spectra from unknown tissue samples using Mahalanobis distance calculations. All mass spectral data belonging to one patient were left out of the sample set, and a new model was calculated using the remaining data. The withheld data were projected into the new model and classified as one tissue type using Mahalanobis distances calculations, performed between the unknown sample point and calculated class centers. This process was repeated for each individual patient. From this, the diagnostic sensitivity and specificity could be calculated as were ROC curves to provide a cross-validated score that describes the clinical utility of the model. To identify statistically significant peaks responsible for prediction into each class, for each cross-validation iteration, analysis of variance (ANOVA) was employed to only select the features most statistically significant (*p* value < 0.05) for PCA-LDA classification into each group. Exact *m/z* values were retrieved from the raw data, and these values were used for compound identification via database search (Metlin).

## Results

Twenty-eight patients were recruited for the ex vivo phase of the study (12 males, 16 females). Two patients were subsequently excluded from the final analysis: In one case, it was not possible to acquire a tissue sample for REIMS analysis because the tumor was not sufficient (a minimum tumor size of 10 mm diameter was required by the histopathologist for research sampling); in the other case, it was not possible to obtain adequate spectra from the REIMS instrument, and the signal-to-noise ratio was too low. Patient demographics and histological classification of the remaining cases are shown in Table 1. Two patients had an adenoma, fifteen patients had adenocarcinoma, eight patients had mucinous adenocarcinoma, and one patient had a gastrointestinal stromal tumor (GIST).

**Table 1:** Patient demographics and histopathological classification from ex vivo analysis

Male/female	11/15
Median age (range)	71 (35–89)
Primary/recurrence	24/2
Neoadjuvant treatment	4
Site of lesion	
Cecum	6
Ascending colon	1
Transverse colon	2
Descending colon	0
Sigmoid colon	7
Rectum	10
Histopathology	
Adenoma	2^a^
Adenocarcinoma	15
Mucinous adenocarcinoma	8
Gastrointestinal stromal tumor (GIST)	1
Tumor differentiation	
Well	1
Moderate	16
Poor	5
Not applicable	4^b^
T stage	
T0	4^b^
T1	1
T2	5
T3	11
T4	5
N stage	
N0	19
N1	6
N2	1
M stage	
M0	25
M1	1
Extramural vascular invasion	7
Lymphovascular invasion	9
Tumor budding	16

^a^ Benign histology but with suspicious features on preoperative imaging

^b^ In addition to the two patients’ tumors which were histologically adenomas, two patients had no viable tumor remaining after complete pathological response to neoadjuvant therapy

## Diagnostic accuracy and histological biomarker association

**Fig. 1:**
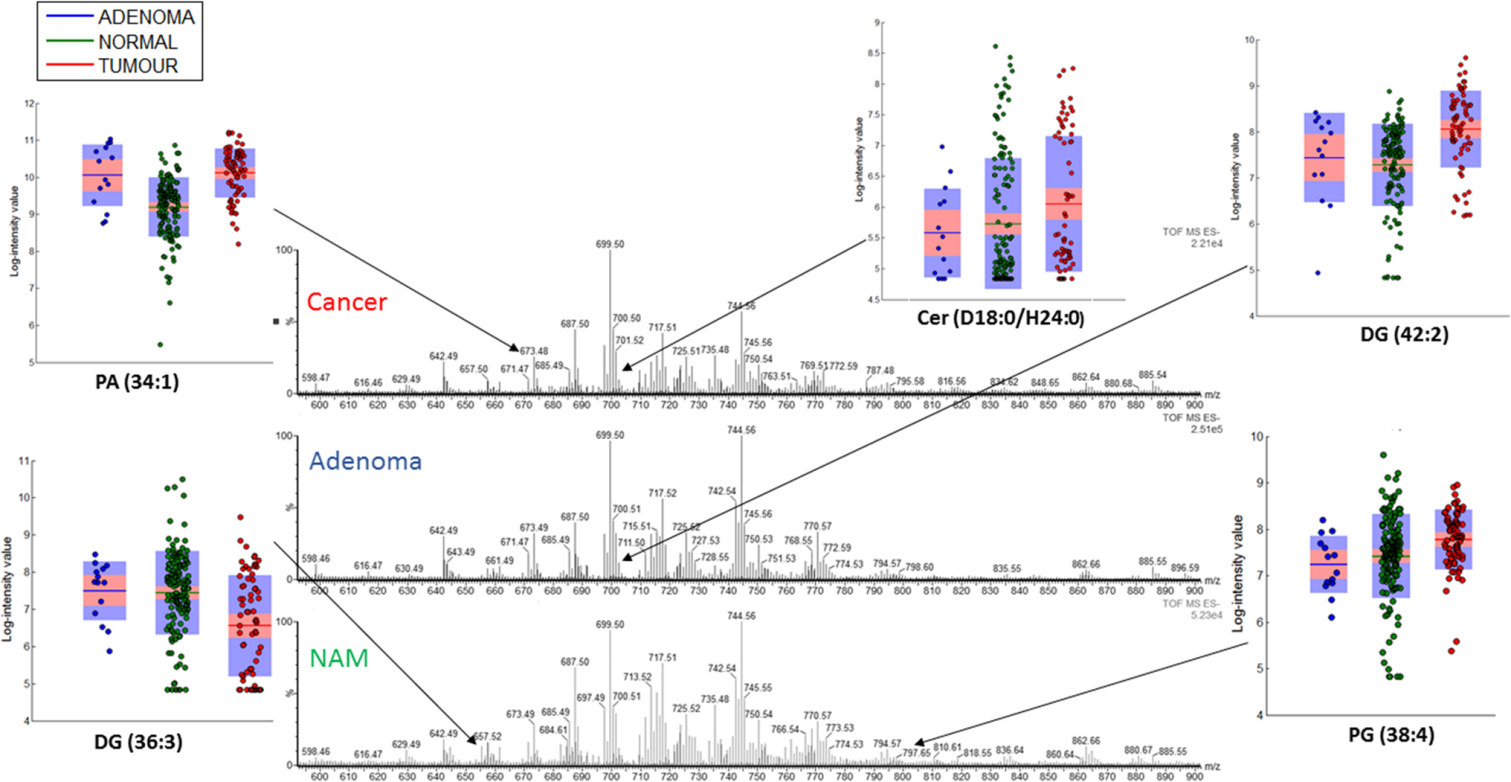
Example spectra from NAM, malignant and adenomatous tissue; the 600–900 m/z range has been selected. *Box plots* representing statistically significant phospholipid species from the entire sample set are demonstrated identifying statistically significant quantitative changes in lipid expression between tissue types

**Fig. 2:**
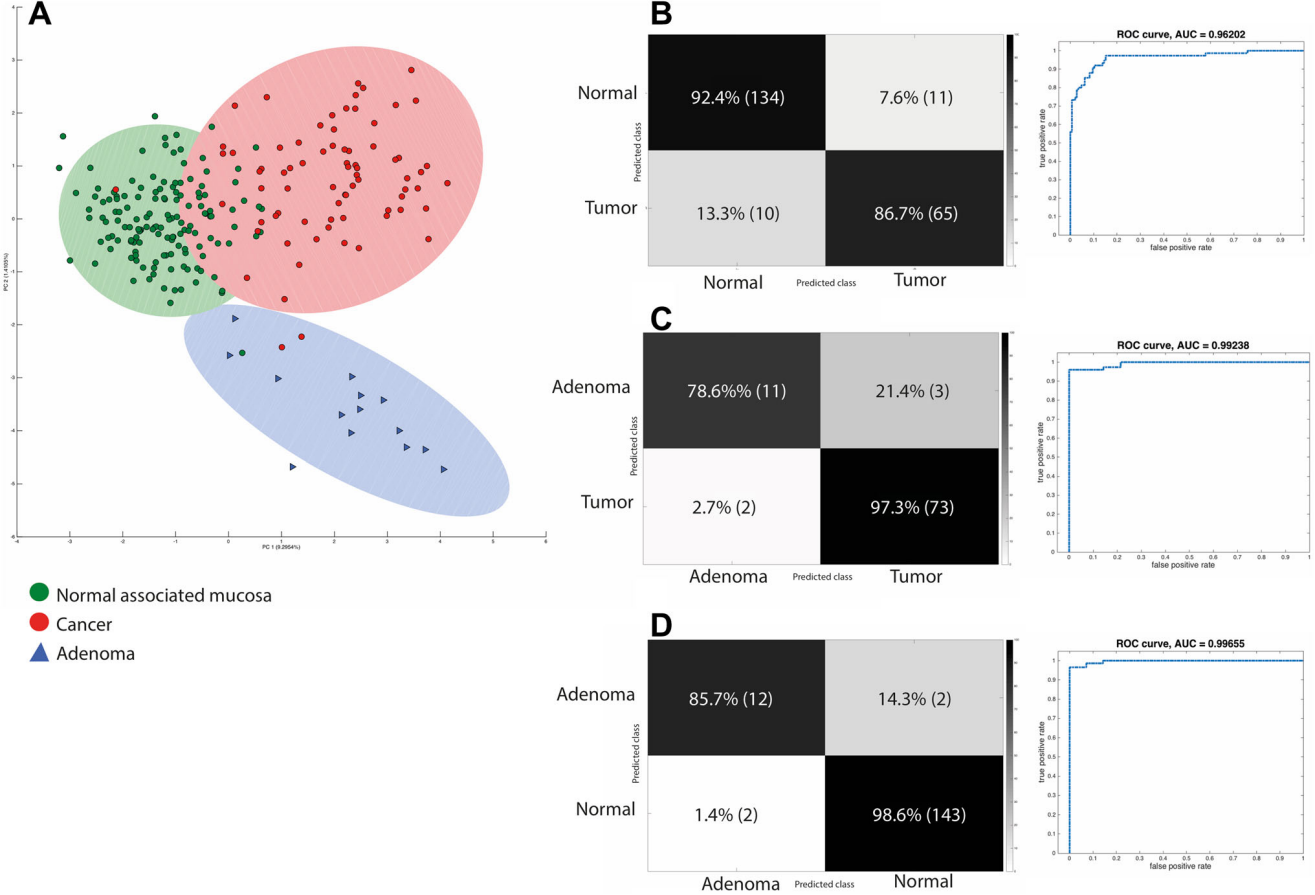
Summary diagnostic colorectal data analyzed by REIMS. **A** An LDA scores plot, demonstrating class separation between normal associated mucosa (NAM), cancer and adenomas. The confusion matrices showing the sensitivity and specificity of **B** NAM versus Tumor **C** Tumor versus adenoma and **D** NAM versus adenoma are demonstrated in the corresponding ROC curves

Representative REIMS spectra from NAM, a cancer and an adenoma can be seen in Fig. 1. REIMS was able to reliably distinguish between NAM and cancer (AUC 0.96) and between NAM and adenoma (AUC 0.99). It had an overall accuracy of 94.4 % for the detection of cancer versus adenoma (Table 2) and an adenoma sensitivity of 78.6 % and specificity of 97.3 % (AUC 0.99) versus cancer (Fig. 2). Univariate analysis of target metabolites was performed based on results from the loadings data to confirm their statistical contribution to the model and to identify descriptive metabolites responsible for class description (Table 3). This confirms that lipid expression in cancer is dynamic, and quantitative changes in target lipids can be identified between pathological disease states. Cancer has an over-expression of long-chain phosphatidylserines (e.g., PS 22:0) and bacterial phosphatidylglycerols, while healthy mucosa has an over-expression of plasmalogens and triacylglycerols (Fig. 1; Table 3), and adenomas demonstrated an over-expression of ceramides (Fig. 1).

**Table 2:** Summary diagnostic and sensitivity data from the multivariate models

	Spectra *n* =	Accuracy	True positive	True negative	False positive	False negative	AUC
Diagnostic markers all							
Cancer versus NAM	220	90.5 %	86.7 %	92.4 %	13.3 %	7.6 %	0.96
Cancer versus adenoma	89	94.4 %	78.6 %	97.3 %	2.7 %	21.4 %	0.99
Adenoma versus NAM	159	97.5 %	85.7 %	98.6 %	1.4 %	14.3 %	0.99
Histological subtype (mucinous vs. adenocarcinoma)	75	90 %	94.2 %	83.3 %	16.7 %	5.8 %	0.96
Prognostic performance—whole model							
Tumor differentiation (mod vs. poor)	183	83.1 %	68.3 %	87.3 %	12.7 %	31.7 %	0.88
Tumor budding	234	78.2 %	80.6 %	74.4 %	25.6 %	19.4 %	0.87
LVI	234	73.9 %	71.6 %	75.3 %	24.7 %	28.4 %	0.83
EMVI	234	73.5 %	65.3 %	77.2 %	22.8 %	34.7 %	0.81
+ve nodes	234	77.4 %	69.0 %	81.0 %	19.0 %	31.0 %	0.81
Rectal cancer prognostic factors							
Differentiation (mod vs. poor)	84	94.4 %	78.6 %	98.2 %	1.8 %	21.4 %	0.99
Tumor budding	84	84.5 %	88.1 %	70.6 %	29.4 %	11.9 %	0.82
LVI	84	71.4 %	72.4 %	30.8 %	69.2 %	27.6 %	0.75
EMVI	84	96.4 %	85.7 %	98.6 %	14.3 %	1.4 %	0.98
+ve nodes	84	92.9 %	83.3 %	94.4 %	5.6 %	16.7 %	0.92
LCRT versus none	75	96 %	95.7 %	96.2 %	3.8 %	4.3 %	0.99
cPR versus NAM	52	100 %	100 %	100 %	0 %	0 %	1

*AUC* area under curve, *NAM* normal adjacent mucosa, *Mod* moderate, *LCRT* long-course chemoradiotherapy, *cPR* complete pathological response

**Table 3:**
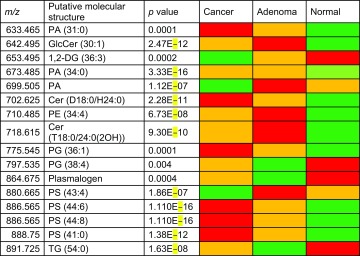
Summary putative metabolite IDs for cancer, adenoma and normal colonic mucosa, their m/z and statistical significance (*p* value)

The array visualization demonstrates if the metabolites were over-expressed (red) or under-expressed (green) in specific histological states of cancer, adenoma or normal associated mucosa

**Fig. 3:**
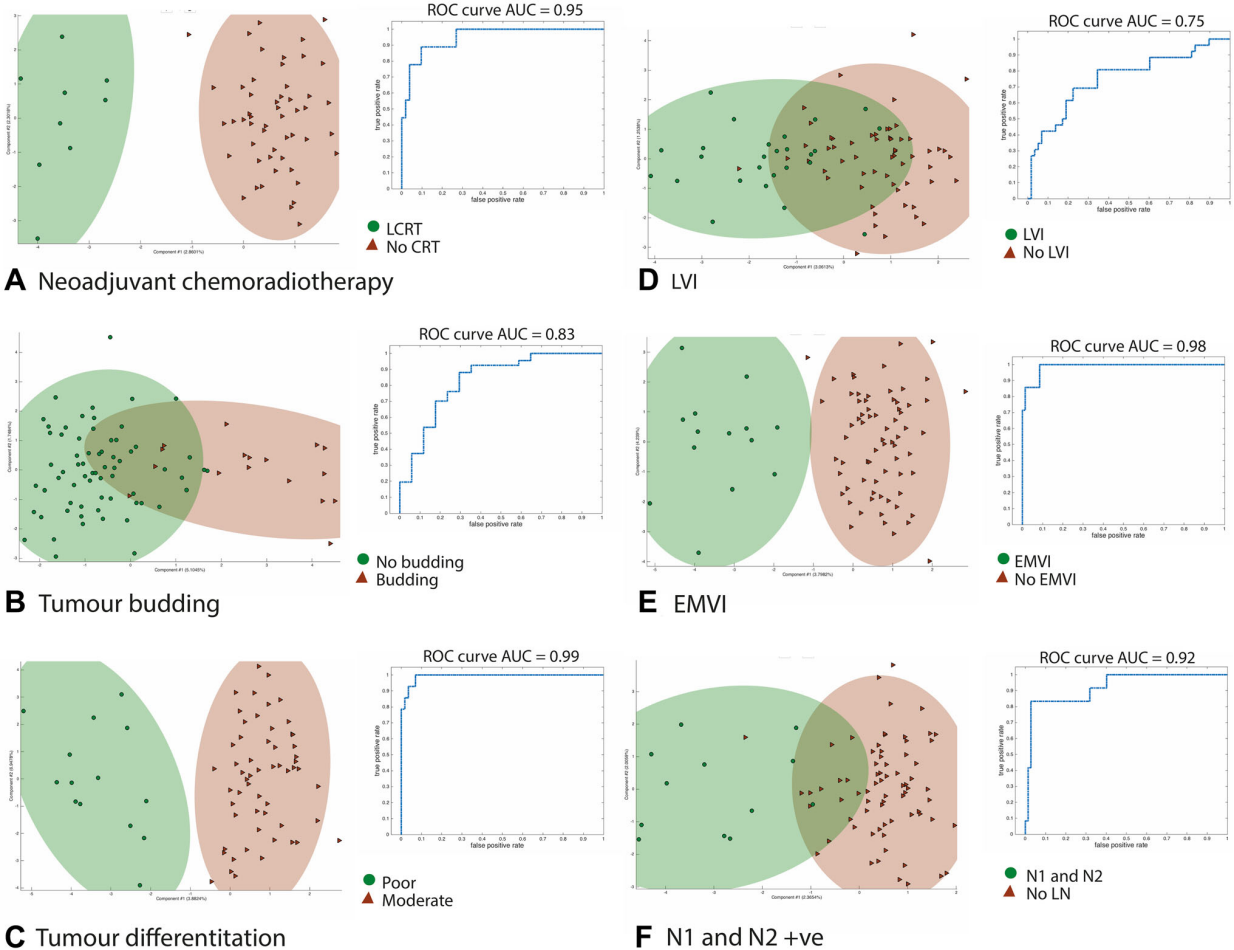
Summary LDA scores plots and associated ROC curves for associations with established histopathological biomarkers of poor prognosis. The model was able to identify those patients that had undergone neoadjuvant LCRT. It was also able to provide robust models for **A** tumor budding **B** differentiation **C** LVI, **D** EMVI and **E** Lymph node micrometastases

To assess the influence of possible patient confounders on the model’s performance, the spectral data were modeled against age and sex, and it was not possible to build a strongly predictive model for either of these factors (Supp. Figure 1 a and b). However, males and patients over the age of 70 did demonstrate discrete clustering, suggesting that there may be both age and sex subtle changes detectable in the colonic mucosa. Analysis of anatomical sampling points also provided a weak model; however, rectal samples were distinctly clustered from the remaining colonic specimens. These data suggest that the rectal mucosa harbors a discrete colonic mucosal lipidome that can be chemically identified (Supp. Figure 1 c).

**Fig. 4:**
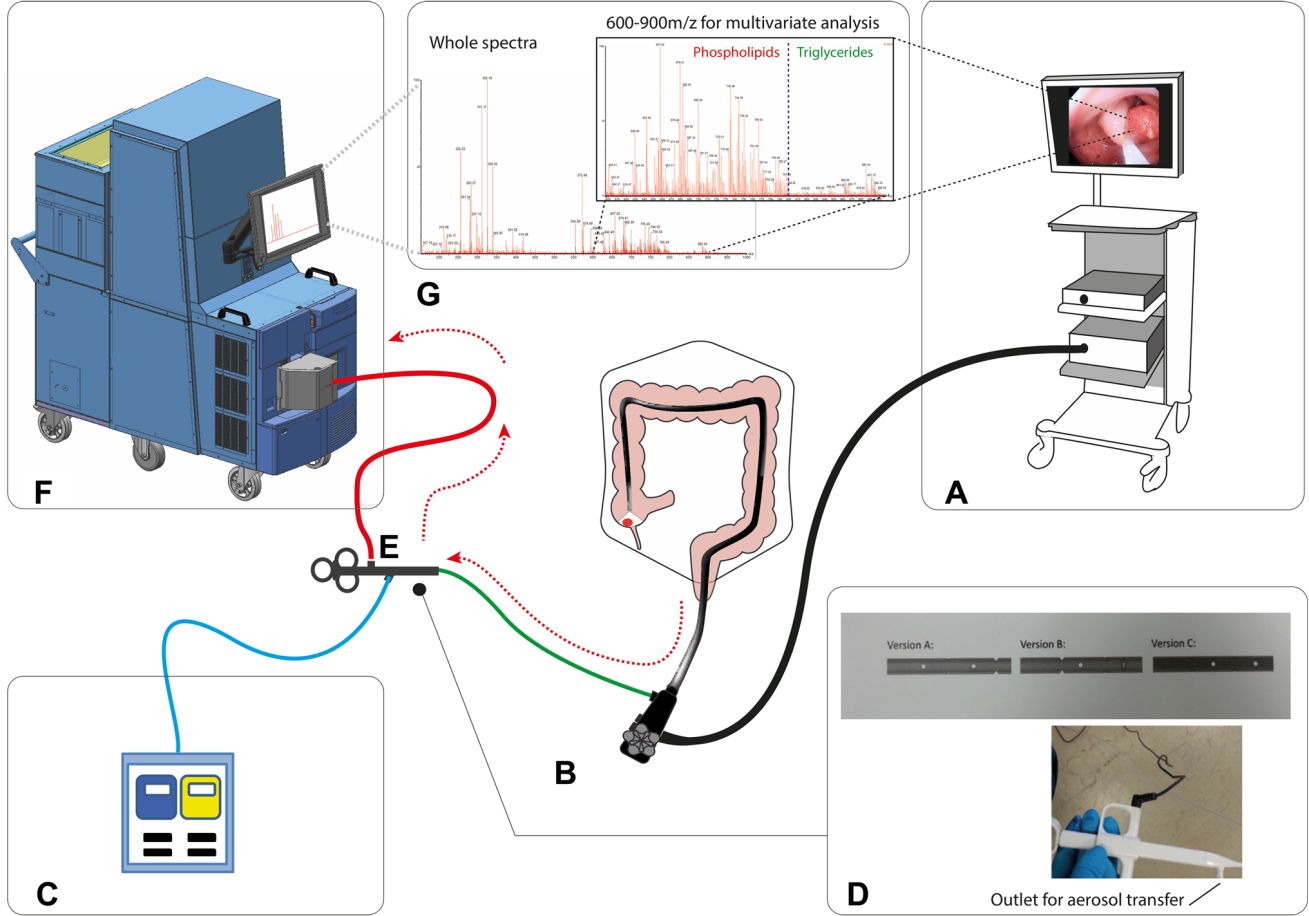
Summary overview of the iEndoscope platform. This technology requires minimal deviation from a standard endoscopic set up with **A** A typical stack and **B** Any commercially available endoscope. **C** A standard electrosurgical generator is deployed during ‘hot’ endoscopic resection. **D** We have created a modified snare with a fenestrated distal sheath for improved efficiency of smoke aspiration. This was developed in three variants. It has a working channel for aspiration directly to **F** a mobile mass spectrometer, with a self contained spectral database sited in the endoscopy suite. Data are in practice displayed as a simple visual readout providing data on predicted histological diagnosis and percentage chances that this is correct. However, the entire spectra are displayed, and the 600–900 m/z range is shown in more detail, as this range is typically used in the multivariate analysis. The predominant phospholipid and triglyceride peaks can be visualized. **G** An adenoma snared during an in vivo assessment of the REIMS platform with representative anatomically discrete lipidomic data captured during hot snare deployment

Using the whole dataset, it was possible to provide information on the histological subtype of tumors. Only one patient in this cohort had a GIST, and although they clustered separately, they were excluded from the analysis. REIMS was able to distinguish between adenocarcinoma and mucinous adenocarcinoma with a high degree of accuracy (Table 2), with an AUC of 0.96 and a sensitivity and specificity of 94.2 and 83.3 %, respectively. As an extension, chemical staging models were constructed to establish how the tumor mucosal lipidome evolves with advancing stage. The overall accuracy of the staging model was 74.7 %, with stage 0 cancers (Fig. 4D) and stage IV cancers clustering separately, and there was considerable overlap between stages II and III lesions. It was possible to classify all tumor specimens according to the lymphatic involvement (Fig. 3) with an accuracy of 83.5 %, although because of the small number of N2 lesions, the sensitivity and specificity was not recordable. The model for metastatic status had sensitivity for distinguishing metastatic from non-metastatic disease of 25 % (Supp. Figure 1 f).

During the in vivo resection of polyps or cancers, numerous sample points are obtained during a single surgical REIMS-augmented procedure, and all of these provide important biochemical data that are relevant to the summary histological classification. Therefore, all sampling points were included (both tumor and NAMs) in the analysis of prognosis. Histopathology revealed that seven tumors had lymph node metastases at the time of histopathological assessment (LN). Seven tumors had extramural vascular invasion (EMVI), and sixteen tumors had tumor budding (TB). The overall performance for all patients included in the analysis was moderate; AUCs of REIMS for all samples in the cohort for differentiation, tumor budding, LVI, EMVI and LN involvement were 0.88, 0.87, 0.83, 0.81 and 0.81, respectively (Fig. 3, Table 2). In general, the specificity of the technique was greater than the sensitivity, although both the false-negative and false-positive discovery rate were variable (Table 2).

To account for the influence on anatomical lipid expression and the confounding effect of neoadjuvant therapies, rectal cancers were then examined in isolation. Four of these patients had undergone neoadjuvant treatment with long-course chemoradiotherapy (LCRT), and multivariate modeling suggested that this has a persistent influence on the rectal lipid chemistry, as the model was able to robustly differentiate those patients treated with LCRT (Sensitivity 95.7 %, specificity 96.2 %, AUC 0.9). Two patients had no viable tumor remaining after complete pathological response to neoadjuvant therapy, however. Histopathological assessment of these biopsies revealed fibrosis, and REIMS was able to robustly distinguish this from healthy tissue with 100 % accuracy (Table 2).

Routine histological reporting outcomes that serve as established biomarkers of poor prognostic outcome and which are used to guide future therapy were then used as arbitrary outcome measures to determine whether REIMS has the ability to define rectal tumors of poor phenotype. REIMS was able to differentiate tumors according to tumor budding (AUC 0.82), differentiation (moderate vs. poor AUC 0.99), LVI (AUC 0.75), EMVI (AUC 0.98) and nodal micrometastases (AUC 0.92). The histological correlations were stronger in the rectal cohort than in the remaining colonic group suggesting that the lipid analysis has the capacity to identify chemical signatures associated with a poor outcome in patients with established rectal cancer.

### In vivo development

The in vivo endoscopic REIMS system was set up as outlined in Fig. 4. A bespoke modified endoscopic polypectomy snare (Medwork) was developed to allow optimum transfer of snare diathermy aerosol from the colonic lumen. Modifications included a series of fenestrations in the distal snare sheath and an inbuilt side port in the snare handle to facilitate connection to the mass spectrometer (Fig. 4D). A video of the system setup and live data capture can be seen in the supplementary data. During the initial experiments, the spectral data were not provided to the endoscopist or clinical team. The system was deployed in vivo in five patients undergoing polypectomy as part of the UK Bowel Cancer Screening Program, and the demographic data for the patients recruited to the in vivo work can be seen in supplementary Table 1. Initial analysis was focused on proof of concept; therefore, the aim was the optimization of data quality, as determined by signal-to-noise ratio. Initial experiments suffered from obstruction of the ion transfer tubing and thus poor quality signals. However, these were overcome, and the snare fenestrations were optimized. An example of the current spectral data quality can be found in the supplementary data (Supp. Figure 3). This demonstrates that target lipid species as identified by the ex vivo analysis are visible within the selected *m/z* charge range, and that they can be captured in near real time. However, it is not currently possible to perform a valid multivariate analysis of its diagnostic strength.

## Discussion

Precision surgery for the management of colorectal cancer requires new technologies that can provide real-time, objective data on tumor biology for the stratification of clinical decision-making. The cancer lipidome is rapidly being realized as a key modulator of cancer biology and therapeutic response, and it provides a novel window into tumor biology. It also offers a novel methodology for in vivo analysis as it can be assessed in near real time. Some tumors demonstrate a lipogenic phenotype, as membrane lipids are rapidly synthesized in tumor cells with a high turnover. Indeed, cancer cells alter lipid metabolism pathways in order to optimize de novo lipid/fatty acid output [11]. Our previous work on colorectal cancer (CRC) metabolism has confirmed markedly altered lipid patterns in healthy compared to cancerous colorectal epithelium [12–15]. In addition, recent cell-based work has implicated lipids as critical regulators of cancer not only structurally (for cell membrane synthesis and stabilization) but also in cell-signaling and cross talk between cancer cells and the cancer-adjacent stroma [11].

This is the first study to apply REIMS to a well-phenotyped, prospectively recruited colorectal cancer patient cohort. The aim of this study was to perform a validation experiment to determine the diagnostic potential of REIMS in colorectal cancer and to investigate the potential of REIMS as a point of care, prognostic stratification tool in colorectal cancer. The technology performed with a high degree of accuracy and was able to robustly distinguish between cancer and NAM, NAM and adenoma and adenomas and cancers. Although promising, the number of adenomatous polyps assessed here was small, and clearly, this requires further analysis in a larger patient population. It has also not assessed whether REIMS is able to detect a focus of cancer within a polyp, which is clearly the gold standard for any technology making an analysis of suspicious polyps. Sphingolipid metabolites have been analyzed by high-performance liquid chromatography (HPLC), and tubular adenomas have been demonstrated to have increased concentrations of ceramides compared to control samples. Ceramide, for example, is characterized by proapoptotic and anti-proliferative properties, and in keeping with the data presented here, its concentration is known to fall with the progression to cancer [16]. This suggests that these data are promising and that REIMS could be used as a tool for the rapid identification of unknown lesions, or for involved in vivo margin detection when electrocautery is deployed during EMR and ESD.

REIMS also demonstrated significant promise in its ability to identify tumors of a poor prognostic phenotype. It was able to consistently identify histopathological features of poor prognosis (tumor budding, differentiation, LVI, EMVI and LN micrometastases). It is therefore possible that REIMS could chemically stage cancers in vivo and that this could be used as an adjunct to standard imaging technologies to improve preoperative staging and for stratifying neoadjuvant therapy. Clearly, we have not demonstrated a mechanistic link between each histopathological feature and target lipids, but this was not the aim of the current study. Moreover, we have not directly compared the performance of REIMS to existing staging methodologies such as MRI, which requires a prospective analysis in a larger patient cohort that we are currently undertaking. What this study demonstrates is that the chemistry of electrosurgical plumes contains rich and detailed information on lesions that are likely to confer a poor prognosis, and these data could therefore be used for stratifying surgical therapy (e.g., radical resection vs. endoluminal therapy and organ preservation). Further work is now needed to identify specific lipid targets and pathways that are regulated by each of these processes. Of specific interest, we were also able to clearly identify rectal samples previously treated with LCRT (Supp. Figure 2). This is not in itself a particular surprise, as radiotherapy will have a significant influence on cell membranes with a high turnover. However, it does suggest that REIMS is able to measure the impact of such an intervention in vivo. If it can be demonstrated that this can be achieved longitudinally, it may serve as a completely novel methodology for predicting complete pathological response in sensitive tumors or for identifying early recurrences.

A number of other optical spectroscopic techniques are being applied to endoscopic characterization of colonic lesions [17, 18]. An advantage of REIMS over existing optical spectroscopic strategies, which relay high-resolution information about lesion structure, is that REIMS provides information about tumor biology and chemistry. For some time, it has been known that increasingly aberrant lipid metabolism is associated with advancing neoplasia [19]. These changes are linked to increasing tumor invasiveness and metastatic potential. In this study, we have shown that by analyzing lipid ratios, REIMS correlates with a series of markers indicative of worsening pathogenicity and thus poorer prognosis in colorectal cancer. The REIMS method can do this in near real time, providing the endoscopist or surgeon with immediate information, which could help to inform decisions about subsequent treatment. This technology is being deployed in vivo and delivering reproducible data. More importantly, it can be deployed as an adjunct to any imaging technology, as the endoscope itself does not require any form of modification. Therefore, REIMS is a tool that can potentially chemically augment other tools deployed in a minimally invasive surgical environment for improving the accuracy of surgical or endoscopic decision-making.

In this analysis, we have gone beyond the ex vivo work to demonstrate a translational application of this precision medicine technology in a first in-man study of colonic polyps. We have successfully used this approach to record viable spectra from patients undergoing elective colonoscopy for polyps. Although this approach has yet to be used for polyp identification, we are able to obtain high-quality spectra that contain many of the metabolites identified from the ex vivo analysis (supp. Figure 3). From this dataset, we are able to generate spectra with an acceptable signal-to-noise ratio, with the level of detail necessary for multivariate analysis. This proof of concept suggests that endoscopic REIMS will be suitable for scaling into an in vivo clinical trial.

The authors acknowledge limitations with this pilot study. The sample size is small, and validation of our results in a larger patient cohort is needed. The exact mechanisms by which REIMS distinguishes clinically relevant variables are also not yet well understood. It is also possible that there is selection bias here as we were only collecting ex vivo specimens at surgery. Normal adjacent mucosa at 10 cm from the primary lesion was available for examination, as opposed to mucosa directly at the lesion margin, in order not to compromise the histopathological integrity of the surgical specimen. Our previous work, using a complementary mass spectrometry technique, suggests that there are metabolic differences between directly adjacent and 10-cm adjacent mucosa [15]. However, the aim of this feasibility study was to assess the diagnostic accuracy of REIMS in detecting cancer, for which 10-cm adjacent mucosa is a suitable control. Energy is not always deployed in the endoscopic biopsy of polyps, and it is not always safe to deploy electrocautery. However, we believe that it is possible to minimize the amount of energy required to sample polyps so that it may be safely used, and these can also be assessed ex vivo after endoluminal capture. Irrespective of this, these data are promising and warrant further examination in the context of a larger-scale study. More importantly, these data will permit power calculations to be performed for future prospective work.

In conclusion, by augmenting optical endoscopic and radiological imaging techniques with information about tissue biochemistry, REIMS offers a new avenue in personalized oncological management through the real-time chemical analysis of the colorectal mucosal lipidome. Larger-scale studies of this technology are now ongoing to try to determine whether the data presented here can be scaled.

## Electronic supplementary material

Below is the link to the electronic supplementary material.

Supplementary material 1 (DOCX 652 kb)

Supplementary material 2 (MP4 87704 kb)
