# Supplementary material for: A novel methodology for in vivo endoscopic phenotyping of colorectal cancer based on real-time analysis of the mucosal lipidome: a prospective observational study of the iKnife
Source: Surg Endosc. 2016 Aug 8;31(3):1361–70. doi: 10.1007/s00464-016-5121-5 (PMC5315709; doi:10.1007/s00464-016-5121-5)

Supplementary Data

| Male/Female | 4/1 |
| --- | --- |
| Median Age (Range) | 67 (61-75) |
| Site of Polyps   - Caecum - Ascending Colon - Transverse Colon - Descending Colon - Sigmoid Colon - Rectum | 1  1  1  0  5  0 |
| Polyp Size   - <5mm - 5-10mm - >10mm | 1  6  1 |
| Polyp Morphology   - Sessile - Pedunculated | 5  3 |
| Polyp Histology   - Hp/LGD/HGD/Ca - TA/TVA/VA | 1/7/0/0  4/3/0 |
| Complete Excision | 8 |

Table 1. Summary patient demographic and histopathology data from the in-vivo analysis. Hp: Hyperplastic; LGD: Low Grade Dysplasia; HGD: High Grade Dysplasia; Ca: Carcinoma; TA: Tubular Adenoma; TVA: Tubullo-villous adenoma; VA: Villous adenoma.

Figure 1. Summary LDA scores plots demonstrating the influence of confounders on the data set. All samples were considered in the analysis of age, sex and anatomical location. There is very limited class separation between a) sex and b) age, although discrete clustering can be observed for males and those patients over the age of 70. c) The model did not provide a strong separation according to anatomical biopsy location, however, rectal samples do cluster separately, and it is also possible to visualize subtle clustering according to the rectum, ascending and descending colon. When only tumour biopsies were analyzed, there was significant overlap of T2 and T3 lesion clusters, although T4 and T0 lesions were metabolically distinct (d). There was also a clear distinction between N0 and N1 and N2 tumours (e), although the model was not able to reliably distinguish metastatic states (f).


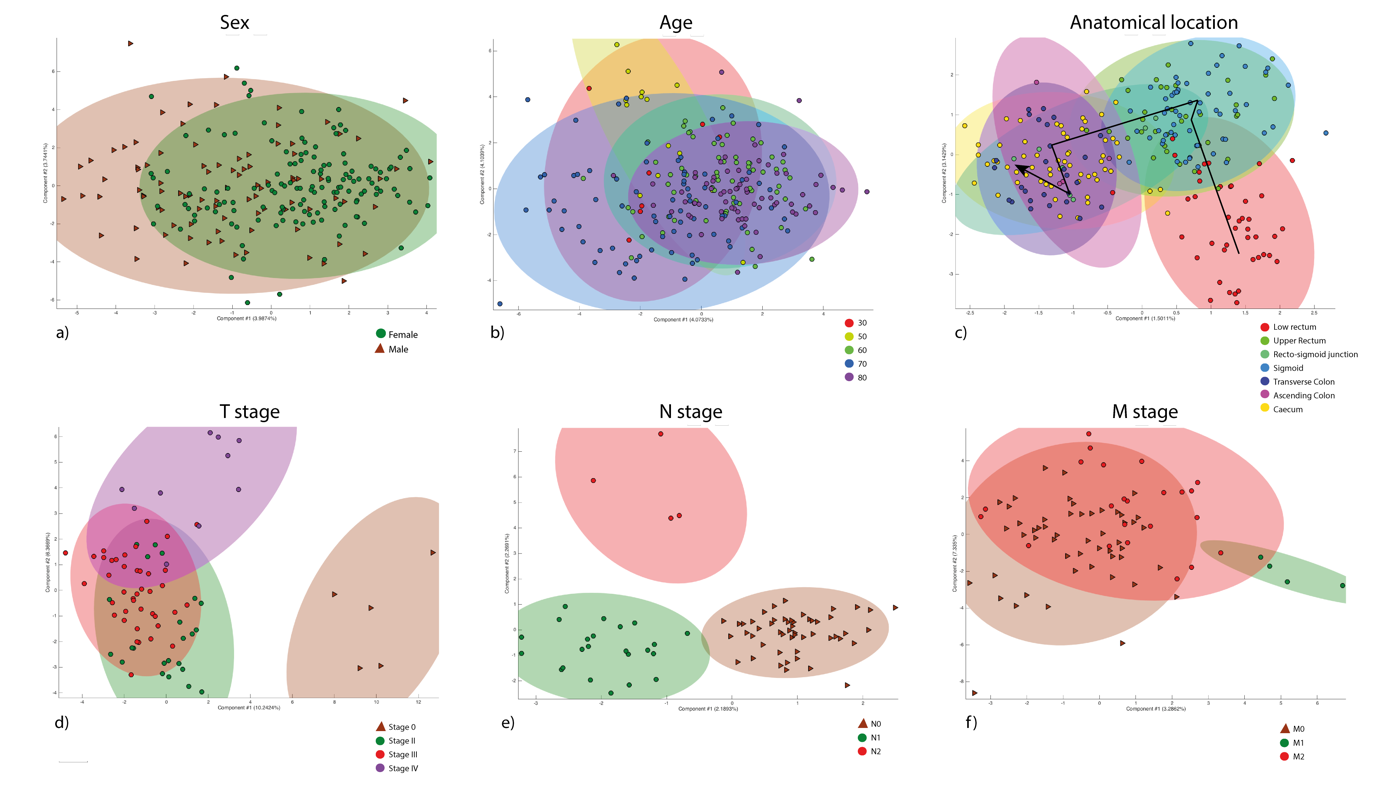


Figure 2. Summary data of spectra taken from patients with a complete pathological response. A) LDA scores plot b) Confusion matrix with diagnostic accuracy c) ANOVA pseudoloadings plot with univariate box plots of statistically descriptive metabolites.


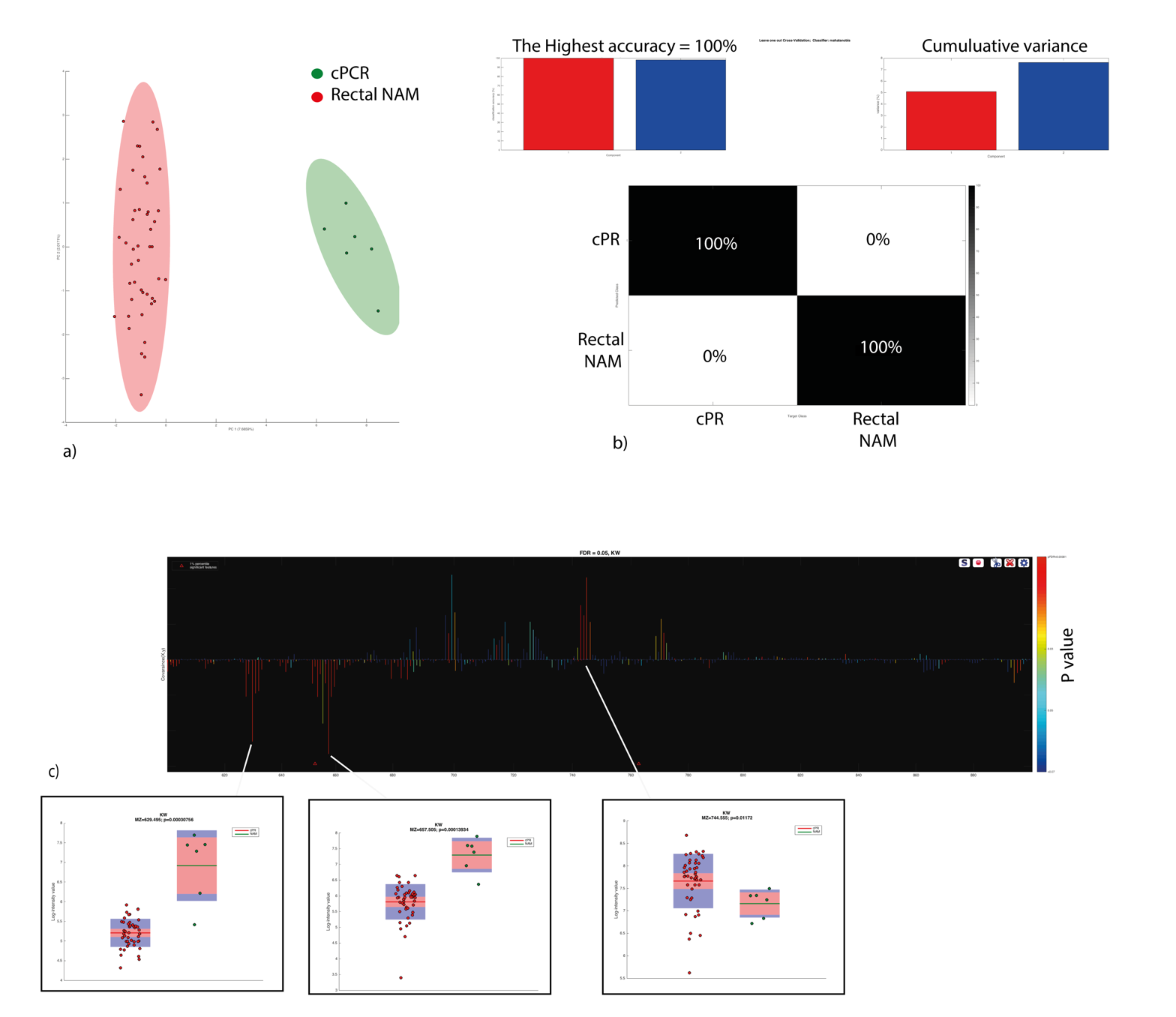


Figure 3. Exemplar spectrum taken from the initial analysis of tissue using the iEndoscope *in vivo* set up.


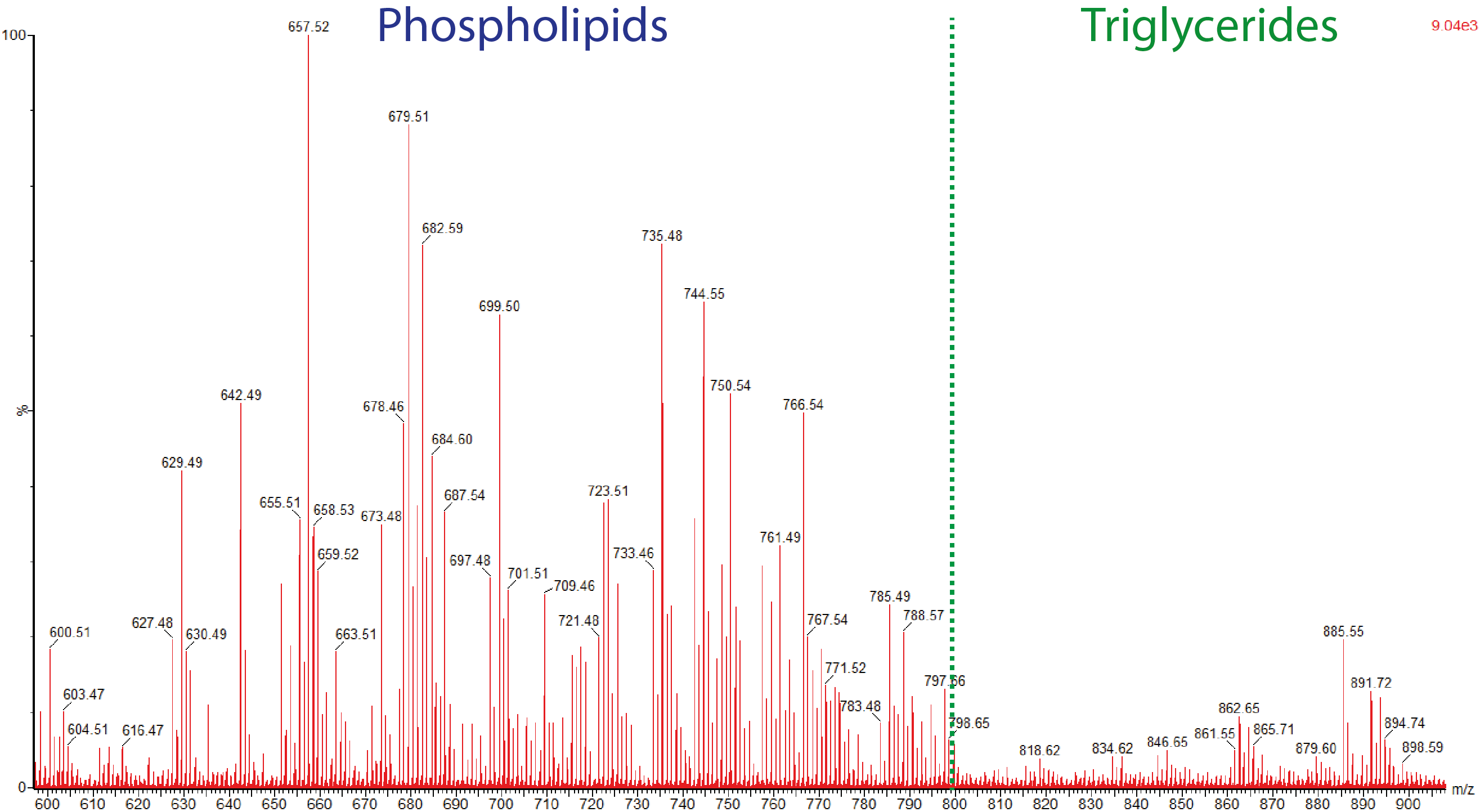

Supplement: Supplementary file 1 — Supplementary material 1 (DOCX 652 kb) [file 464_2016_5121_MOESM1_ESM.docx]
